# From Marine Origin to Therapeutics: The Antitumor Potential of Marine Algae-Derived Compounds

**DOI:** 10.3389/fphar.2018.00777

**Published:** 2018-08-06

**Authors:** Celso Alves, Joana Silva, Susete Pinteus, Helena Gaspar, Maria C. Alpoim, Luis M. Botana, Rui Pedrosa

**Affiliations:** ^1^MARE – Marine and Environmental Sciences Centre, ESTM, Polytechnic Institute of Leiria, Peniche, Portugal; ^2^Biology Department, Do^*^Mar Doctoral Programme on Marine Science, Technology and Management, University of Aveiro, Aveiro, Portugal; ^3^Faculty of Sciences, BioISI - Biosystems and Integrative Sciences Institute, University of Lisboa, Lisbon, Portugal; ^4^Faculty of Sciences and Technology, University of Coimbra, Coimbra, Portugal; ^5^Center of Investigation in Environment, Genetics and Oncobiology, University of Coimbra, Coimbra, Portugal; ^6^Center for Neuroscience and Cell Biology, University of Coimbra, Coimbra, Portugal; ^7^Departament of Pharmacology, Faculty of Veterinary, University of Santiago de Compostela, Lugo, Spain

**Keywords:** cancer, marine natural products, seaweeds, intracellular signaling pathways, biodiversity, marine chemical ecology, *Sphaerococcus coronopifolius*

## Abstract

Marine environment has demonstrated to be an interesting source of compounds with uncommon and unique chemical features on which the molecular modeling and chemical synthesis of new drugs can be based with greater efficacy and specificity for the therapeutics. Cancer is a growing public health threat, and despite the advances in biomedical research and technology, there is an urgent need for the development of new anticancer drugs. In this field, it is estimated that more than 60% of commercially available anticancer drugs are natural biomimetic inspired. Among the marine organisms, algae have revealed to be one of the major sources of new compounds of marine origin, including those exhibiting antitumor and cytotoxic potential. These compounds demonstrated ability to mediate specific inhibitory activities on a number of key cellular processes, including apoptosis pathways, angiogenesis, migration and invasion, in both *in vitro* and *in vivo* models, revealing their potential to be used as anticancer drugs. This review will focus on the bioactive molecules from algae with antitumor potential, from their origin to their potential uses, with special emphasis to the alga *Sphaerococcus coronopifolius* as a producer of cytotoxic compounds.

## Introduction

Natural products (NPs) have been used as therapeutic agents for the treatment of a wide spectrum of illnesses for thousands of years, playing an important role in meeting the basic needs of human populations. In 1985, World Health Organization estimated that ~65% of world population ensured their primary health care using predominately plant-derived traditional medicines, existing a less prevalence in developed countries (Cragg and Newman, 2013). Due their unusual chemical features, NPs have functioned as scaffolds for the development of new products with huge therapeutic and industrial potential. Moreover, these compounds present a greater efficiency and specificity with target sites since were originated in co-evolution with biological systems. These interesting compounds result from the interactions between organisms and their environment, which promote the production of diverse complex chemical compounds by the organisms to increase their survival and competitiveness (Mishra and Tiwari, 2011).

Comparing with terrestrial organisms, marine organisms do not have a distinguished history of use in traditional medicine. However, in the last 50 years, advances in new technologies and engineering such as scuba diving techniques, manned submersibles and remotely operated vehicles (ROVs) opened up the marine environment to scientific exploration (Cragg and Newman, 2013). The coexistence of several species in these habitats of limited extent increases their competitiveness and complexity. For example, sessile organisms such as algae, corals, sponges, and other invertebrates are in constant competition and many of them have evolved chemical means to defend themselves against predation or overgrowth of competing species or, conversely, to subdue motile prey species for ingestion. These chemical adaptations are generally defined as “secondary metabolites” and involve different classes of chemical compounds, which have evidenced great pharmacological potential (Simmons et al., 2005). Therefore, marine organisms have revealed to be an exceptional reservoir of NPs, some of them with different structural features from those of terrestrial sources. Despite considerable challenges, some marine compounds arrived in the market and are currently used in therapeutics, providing a useful roadmap for future translational efforts (for details, please see topic 4; Arizza, 2013). Among the different illnesses, cancer is a growing threat to public health, particularly in developed countries, and it is expected that their occurrence and associated deaths will increase in the next years (American Cancer Society, 2015). This problem is directly associated with the growth and aging of the population and the adoption of behaviors that contribute to increase cancer risk (American Cancer Society, 2015). Moreover, owing to the tumor cells resistance to drugs, significant toxicity, and undesirable side effects observed with synthetic drugs, there is an urgent need for new antitumor drugs development (Sawadogo et al., 2015; Torre et al., 2015). Given that cancer is a multifactorial and multi-targeting disease that cannot be prevented by mono-targeted therapies, many researchers have focused their efforts toward NPs, especially those from marine environments, to identify novel anticancer compounds. Today, it is estimated that more than 60% of anticancer drugs presently in the market are of natural origin (Cragg and Newman, 2009). In addition, there are several natural compounds originated or derived from marine life presently undergoing clinical trials with oncological indications (AndisInsight, 2018; Calado et al., 2018; EMA, 2018a,b; FDA, 2018a,b; Mayer, 2018).

This review contains six topics condensing the importance of marine resources as source of antitumor compounds, summarizing the most important works accessing the potential of algae as source of marine drugs for cancer therapeutics.

## Cancer biology—general overview

Cancer is one of the major human health problems worldwide, with high social and economic impacts. There is evidence of this disease in antiquity, dating back to the times of the Pharaohs in ancient Egypt and the classical world (Nobili et al., 2009). Currently, worldwide, cancer is responsible by one in each seven deaths causing more deaths than AIDS, tuberculosis and malaria combined (American Cancer Society, 2015). Only surpassed by cardiovascular diseases, cancer is the second leading cause of death in high-income countries while being the third leading cause of death in low- and middle-income countries following cardiovascular, infectious and parasitic diseases (American Cancer Society, 2015). By 2030, it is estimated that the incidence of this illness grows to over 21.7 million new cases and 13 million deaths (American Cancer Society, 2015). Beyond the social impact, cancer is also associated with high financial costs at the individual and social level for both the person with cancer and for society as a whole. For example, in 2011 in the US the direct medical costs (total of all health care expenditures) associated with cancer was estimated in $88.7 billion, which are related with hospital outpatient or office-based provider visits, patient hospital stays and medical prescriptions (American Cancer Society, 2015).

However, what we simply call “human cancer” comprises, in fact, more than 100 different diseases that result from the continuous uncontrolled proliferation of cancer cells (Urbano et al., 2011), which have the capability to invade organs and normal tissues, as well as metastasizing through the body. These cells do not respond properly to the signals that regulate their normal behavior (Schulz, 2007; Cooper and Hausman, 2013). In line with this view, Hanahan and Weinberg (2000) published a review article that combined information about cancer biology and defined six hallmarks (sustaining proliferative signaling, resisting cell death, inducing angiogenesis, enabling replicative immortality, activating invasion and metastasis and evading growth suppressors) that all cancer cells have and that are responsible for their malignant properties. Subsequently, an upgrade of this list was done adding two new hallmarks, deregulating cellular energetics and evading immune destruction. Nevertheless, the occurrence of these hallmarks is directly associated with the genome instability, which is responsible by genetic diversity that stimulates their acquisition, and inflammation and promotes multiple hallmark functions (Hanahan and Weinberg, 2011). Recently, case–control metagenomics studies suggest that dysbiosis in the commensal microbiota is also associated with various cancer types adding microbiome as an additional hallmark (Rajagopala et al., 2017). The complexity of tumors represents a great challenge for therapeutic approaches, as experimental evidence exists that each core hallmark capability is regulated by partially redundant signaling pathways (Hanahan and Weinberg, 2011). Consequently, targeted therapy mediated by drugs that only act on one key pathway in a tumor may not be enough to “switch off” a hallmark capability completely. As a result some cancer cells can survive maintaining a basal function awaiting an adaptation of their progeny to the selective pressure imposed by the drug. This adaption can be accomplished by genetic changes, epigenetic reprogramming, or remodeling of the stromal microenvironment. All of these processes can contribute for restitution of the functional capability, allowing renewed tumor growth and consequently clinical relapse (Hanahan and Weinberg, 2011). Nevertheless, the drug resistance of tumor cell lines can also be mediated by other mechanisms, such as drug efflux, suppression of drug activity, changes in cellular targets, enhanced DNA repair, inability to induce cell death and the epithelial-mesenchymal transition (Housman et al., 2014). Among all the treatments currently used in cancer (surgery, radiotherapy, hormonal treatment and immunotherapy, adjunct therapy, and chemotherapy), chemotherapy continues to play an extremely important role. However, its effectiveness is limited in some cases by the existence of drug resistance, making it necessary to define optimal combinations for therapeutic strategies that ensure an efficient elimination of the tumor. Moreover, in the last decades, with the continuous growth of cancer cases and concerns over toxicity, tumor cell resistance, the development of secondary cancers and the unwanted side effects observed with synthetic drugs, there has been an increased interest in exploiting NPs for cancer treatment (Newman and Cragg, 2012; Sawadogo et al., 2015).

## Role of the marine chemical ecology in the production of bioactive metabolites on algae

The oceans represent a vast area of the planet and play a fundamental role in its dynamic. Their physics, chemistry and biology are key elements in the functioning of the earth system, providing an interconnection between the different natural systems (terrestrial, freshwater, estuarine, coastal, and oceanic) and a range of valuable ecosystem services (Atkins et al., 2011; Halpern et al., 2012; Botana and Alfonso, 2015). Their essential role is further noted by the significant fraction of the Earth's biodiversity that oceans harbor (Brahmachari, 2015). According to the 33 animal phyla listed by Margulis and Chapman (2009), 32 of them are represented in aquatic environments, with 15 exclusively marine, 17 found in marine and non-marine environments (with 5 of these having more than 95% of their species only in marine environments), and only one exclusively non-marine (*Onychophora*). A recent study predicted the existence of ~8.9 million eukaryotic species, of which ~2.2 million are marine organisms, suggesting that around 86% of the species on the earth, and 91% in the ocean, have not yet been described (Mora et al., 2011; Cragg and Newman, 2013; Berkov et al., 2014). The existence of a huge diversity of life forms in the oceans is associated with the very exigent, competitive and aggressive surrounding that promotes specific and complex interactions, both inter-species and intra-species. During the evolutionary period, many species share a common environment establishing well-balanced associations between them. Inside of these communities several organisms survive and live in close association with other species, both macro (e.g., algae, sponges, and ascidians) and micro (e.g., bacteria, fungi, and actinomycetes) in order to ensure their survival (Da Cruz et al., 2012; Graça et al., 2013; Horta et al., 2014; Smith et al., 2018). Many of these complex interactions are mediated by chemical signals, which play a crucial role at the organizational level in the marine environment (Hay, 2009). These chemical cues constituting much of the language of sea life and are the utmost importance for several marine species which have not some senses such as vision and hearing; nevertheless even species that see and hear rely on chemical cues (Botana and Alfonso, 2015). Interactions mediated by chemical signals play a crucial and decisive role in ecological processes. These signals influence population structure, community organization and ecosystem function. In addition they are involved in the definition of escape strategies, commensal associations, partners and habitats, competitive interactions, feeding choices and energy and nutrients transfer within and among ecosystems (Hay, 2009).

Among marine organisms, algae are a clear example in which chemical signals play a fundamental role in ecological processes. These signs are involved in the growth and survival in extremely exigent conditions, giving them competitive advantages relative to other marine organisms including against predators and competitors. One such piece of evidence has recently been observed in coral reefs, which are in dramatic global decline, with algae commonly replacing them. Several studies have observed that algae damage corals directly or colonize opportunistically, suppressing coral recruitment through the production of specific chemical cues repulsing the recruits (Rasher and Hay, 2010, 2014; Dixson et al., 2014). Recently, Rasher et al. (2011) identified four compounds (two loliolide derivatives and two acetylated diterpenes) from two algae as potent allelochemicals which directly damage corals. Marine organisms sometimes face the dilemma of how to allocate the limited resources available, having to strategically adapt. Usually these decisions may have consequences on their growth, reproduction, or ability to counteract biological (e.g., predators, maintenance of unfouled surfaces, paralyzing their prey, etc.) and/or physical stress (e.g., UV light, temperature, nutrient availability, high pressure, salinity, oxygen content, etc.) (Winter et al., 2010; Botana and Alfonso, 2015). The production of these types of compounds has also been revealed to be an important weapon for the successful invasion of non-indigenous species into new ranges. For instance, the invasive red alga *Bonnemaisonia hamifera* has become one of the most abundant species in Scandinavian waters. According to Svensson et al. (2013) the high capacity of this alga to colonize these waters seems to be linked with the presence of a specific chemical compound (1,1,3,3-tetrabromo-2-heptanone). The production of this metabolite inhibit the settlement of propagules on its thallus and on surrounding surfaces, achieving a competitive advantage over native algae (Svensson et al., 2013). Chemical cues also play an important role in the symbiotic interactions established between algae and microorganisms. The cross-kingdom interactions between them are not restricted to the exchange of macronutrients, including vitamins and nutrients but also include the use of infochemicals with different functions, establishing a tight relationship and enabling them to interact as a unified functional entity (Egan et al., 2013; Wichard, 2015). For instance, associated microorganisms are responsible to produce compounds of utmost importance which mediate essential ecological functions in the development and growth of algae species including quorum sensing signaling molecules, compounds with biological activities, substances that promote the growth and other effective molecules compounds (Singh and Reddy, 2014). Some of these compounds, such as bacterial morphogenetic compounds, dimethylsulfoniopropionate (DMSP), the amino acids proline and alanine, halogenated furanones and fucoxanthin, play important roles in the ecological function, interfering with the surface fouling of others organisms as well as with the vital functions performance of the algae. These compounds prevent the attachment of certain bacteria (e.g., *Cytophaga* sp.) and support the fixation of others (e.g., *Rheinheimera baltica*), controlling the community composition and abundance of the algae-associated bacteria (Saha et al., 2011, 2012; Spoerner et al., 2012; Egan et al., 2013). Marine organism interactions promote the production of a high diversity of marine NPs with quite specific and potent activities, representing an enormous source of new compounds with potential for biotechnology applications providing economic and human benefits.

## Marine natural products as a source of new drugs and current clinical pipeline

Along of the evolution, marine organisms developed exceptional metabolic capacities through the production of compounds with quite specific and potent activities (Murray et al., 2013; Martins et al., 2014). These compounds, often defined as secondary metabolites, are generally limited to a particular taxonomic family, genus, species or even organism, characterized by their wide heterogeneity, and often constitutes a very small fraction of the total biomass of the organism (Ianora et al., 2006; Avila et al., 2008; Martins et al., 2014). Predominantly, their production occurs in sessile or slow-moving organisms (e.g., algae, sponges, cnidarians, tunicates and bryozoans) that, without effective escape mechanisms or structural protection, ensure their protection through chemical defense (Noyer et al., 2011; Botana and Alfonso, 2015). Nevertheless, many organisms have the capacity to sequester secondary metabolites from their diet and then derivatize them to more or less toxic forms can be these used for functions different from their roles in the original producer (Ianora et al., 2006; Kicklighter et al., 2011; Botana and Alfonso, 2015; Gotsbacher and Karuso, 2015). Moreover, since natural compounds released into the water are rapidly diluted, they need to be highly potent to retain their efficacy (Haefner, 2003). For these reasons, it is widely accepted that a huge number of NPs and novel chemical entities that exist in the oceans could be useful for providing sustainable economic and human benefits.

To date, different types of secondary metabolites (e.g., terpenoids, alkaloids, polyketides, peptides, shikimic acid derivatives, sugars, steroids, and a large mixture of biogenesis metabolites) were isolated from marine organisms and found to exhibit many biological activities (antimicrobial, antitumor, antidiabetic, anticoagulant, antioxidant, anti-inflammatory, antiviral, antimalarial, antitubercular, anti-aging antifouling, and antiprotozoal) with huge industrial and therapeutic potential (Blunt et al., 2013, 2014; Mayer et al., 2013, 2017; Agrawal et al., 2018). Marine NPs have exhibited rare and unique chemical structures, upon which the molecular modeling and chemical synthesis of new drugs can be based on (Dias et al., 2012; Botana and Alfonso, 2015). Since this kind of compounds are originated from nature present several advantages compared with synthetic compounds such as a chemical diversity, biochemical specificity, binding efficiency, and affinity to interact with biological systems, making them interesting structures for development of new drugs (Martins et al., 2014).

**Figure 1:**
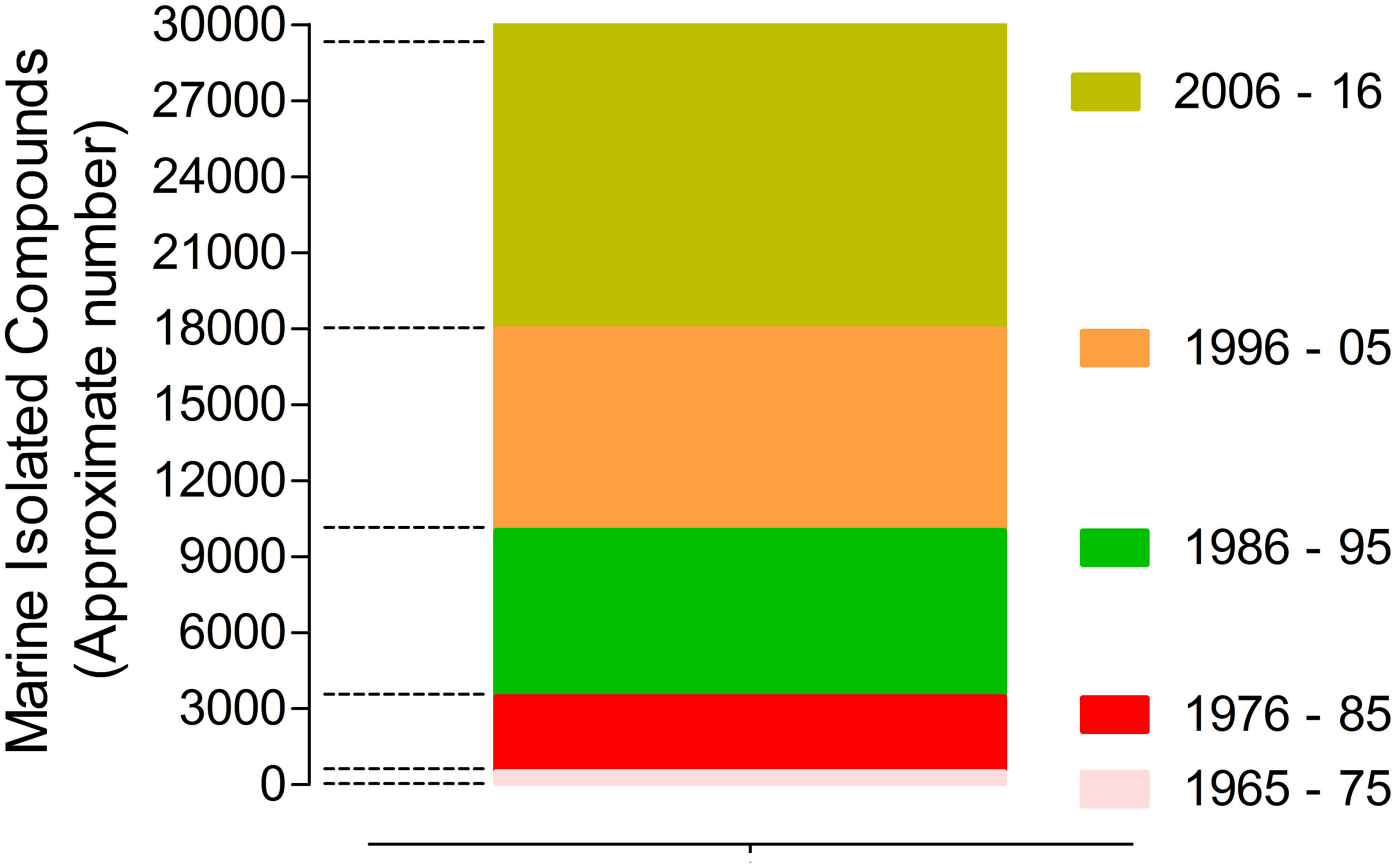
Marine compounds isolated in the last 50 years (approximate number/10 years) (Faulkner, 1984, 1986, 1987, 1988, 1990, 1991, 1992, 1993, 1994, 1995, 1996, 1997, 1998, 1999, 2000, 2001, 2002; Blunt et al., 2003, 2004, 2005, 2006, 2007, 2008, 2009, 2010, 2011, 2012, 2013, 2014, 2015, 2016, 2017, 2018).

The marine biodiscovery and vision of marine-derived drugs on the market had their beginning in the early 1950s with Bergmann, who isolated and identified two nucleosides, spongouridine and spongothymidine, from the Caribbean sponge *Cryptotethya crypta* (previously known as *Tethya crypta*). These discoveries led researchers to synthesize analogs, *Ara-A* (*Vidarabine*®, *Vidarabin Thilo*®) and *Ara-C* (*Cytarabine, Alexan*®, *Udicil*®), the first marine derived compounds that have reached the market as antiviral and antitumor drugs, respectively, (Newman et al., 2009; Botana and Alfonso, 2015). Over the last 50 years was reported the isolation of more than 30,000 new compounds of marine origin (Figure 1) and the approval of more than 300 patents (Blunt et al., 2015; Botana and Alfonso, 2015).

Nevertheless, despite of the remarkable potential of marine NPs as source of new drugs their role have undergone several changes, having had an evident decline in the pharmaceutical R&D activities by the mid-1990s. After that decline, the larger research and development effort in the exploration of this niche was essentially assured by enterprising academics, mainly partnered with industry. In the last decade, this area seems to have benefited from a renaissance, since the number of new isolated marine compounds has increased when compared with the previous similar period (Molinski et al., 2009; Botana and Alfonso, 2015). This rebirth is directly associated with recent technological advances in analytical technology, spectroscopy, and high-throughput screening. Advances in “omics” techniques (genomics, metagenomics, proteomics), combinatorial biosynthesis, synthetic biology, selection methods, expression systems, and bioinformatics have contributed as powerful tools to discover new chemical entities with pharmaceutical potential (Molinski et al., 2009; Bucar et al., 2013).

**Figure 2:**
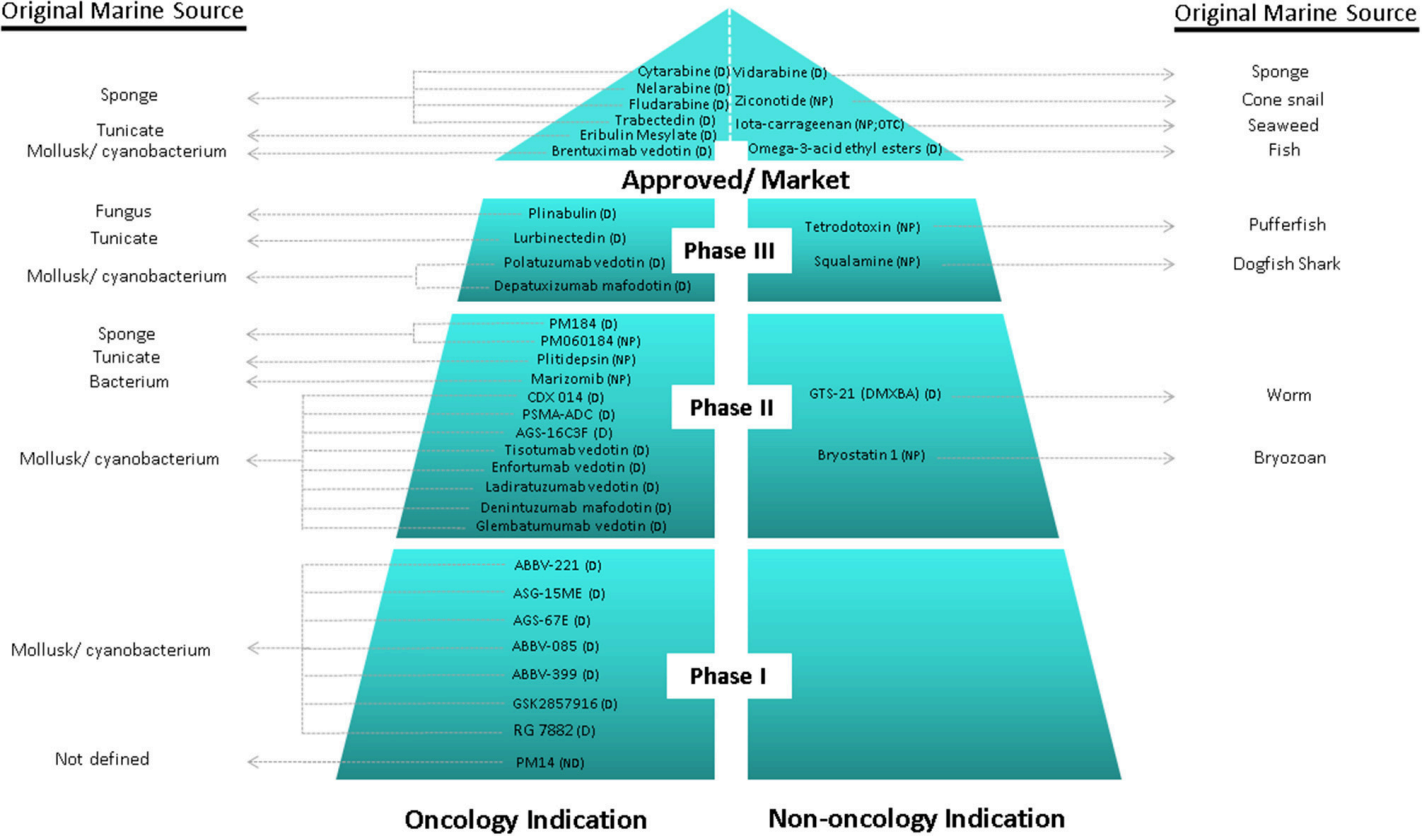
Current clinical pipeline of marine-derived drugs and their original marine source. NP, Natural product; D, Derivate; OTC, Over-the-counter (AndisInsight, 2018; Calado et al., 2018; EMA, 2018a,b; FDA, 2018a,b; Mayer, 2018).

Over the last 30 years, great efforts have been made, showing productive and promising results, since it has been defined the major tendencies in secondary metabolism of several classes of marine organisms. Only in the last 20 years, more than 18,000 new marine compounds were described and six (Figure 2) out of the nine marine-derived drugs currently used in clinical therapy were approved, as well as one over-the-counter drug (OTC). Cytarabine (Cytosar-U®), Vidarabine (Vira-A®) (US discontinued), Ziconotide (Prialt^®;^), Brentuximab Vedotin (Adcetris®), Eribulin Mesylate (Halaven®), Omega-3-acid ethyl esters (Lovaza^®;^), Trabectedin (Yondelis®), Fludarabine Phosphate (Fludara®), and Nelarabine (Arranon®) were approved by the Food and Drug Administration (FDA) in the US Pharmacopeia and/ or by the European Agency for the Evaluation of Medicinal Products (EMA). Iota-carrageenan (Carragelose®), one OTC, was approved by EMA. However, it is expected that the number of newly approved drugs from marine origin will continue to increase, since 28 marine or marine-derived drugs are currently in clinical trials (six marine molecules—phase III; fourteen marine molecules—phase II; eight marine molecules—phase I) (Figure 2) (AndisInsight, 2018; Calado et al., 2018; EMA, 2018a,b; FDA, 2018a,b; Mayer, 2018).

**Figure 3:**
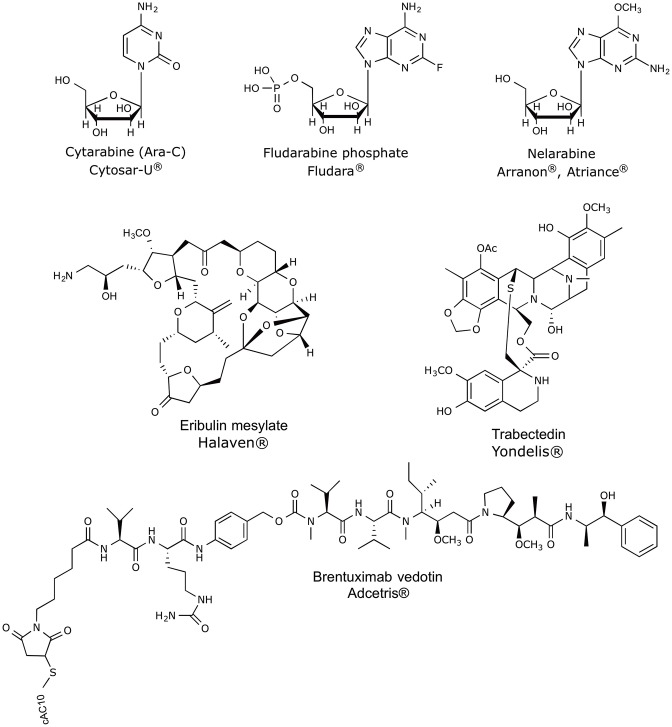
Chemical structures of anticancer marine-derived drugs in the market.

Considering all the marine drugs available in the market, it is particularly interesting to see that six are used in cancer therapies (Figure 3), and the majority of the compounds that are in clinical trials are also for application in cancer therapy, which reveals the great potential of marine compounds as anticancer drugs (Figure 2).

Although not associated with cancer therapeutics, iota-carrageenan (Carragelose®) is the first product developed from algae in the market. It is a type of carrageenan, isolated from a red edible algae belonging a family of linear sulfated polysaccharides. Carragelose® has ability to block viral attachment to the host cells being effective against a broad spectrum of respiratory viruses (Ludwig et al., 2013; Calado et al., 2018).

## Antitumor potential of marine algae-derived compounds

**Figure 4:**
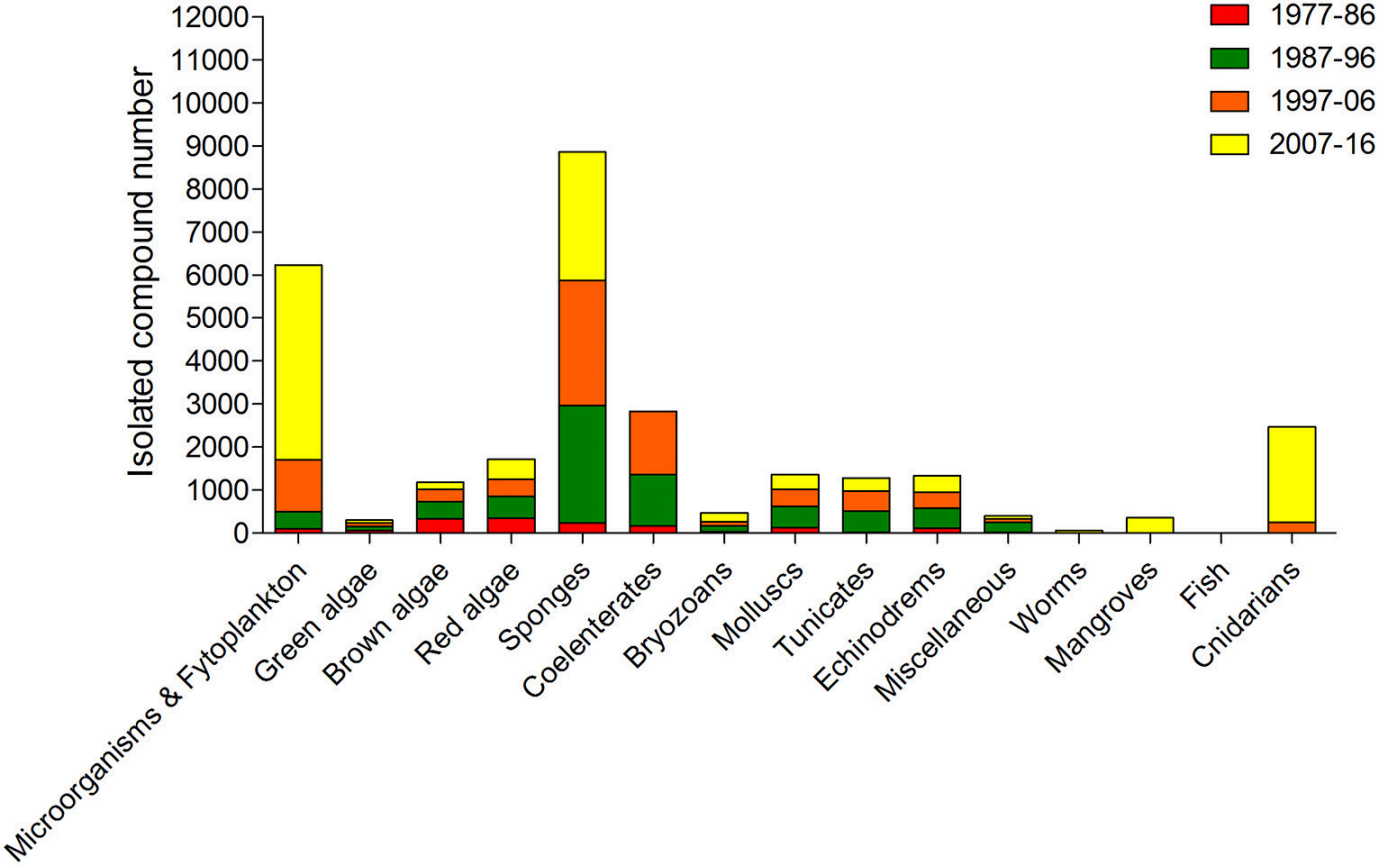
Approximate numbers of new compounds isolated from different marine organism sources between 1977 and 2016 (Faulkner, 1984, 1986, 1987, 1988, 1990, 1991, 1992, 1993, 1994, 1995, 1996, 1997, 1998, 1999, 2000, 2001, 2002; Blunt et al., 2003, 2004, 2005, 2006, 2007, 2008, 2009, 2010, 2011, 2012, 2013, 2014, 2015, 2016, 2017, 2018).

Among marine organisms, algae are one of the most important resources of the ocean, economically and ecologically (Kim, 2014). Their inclusion in the Asian diet has been associated with health benefits, where there has been observed a lower incidence of chronic diseases, such as hyperlipidaemia, coronary heart disease, diabetes and cancer, according to epidemiological studies comparing Japanese and Western diets (Brown et al., 2014; Bouga and Combet, 2015). Algae are valuable sources of protein, fiber, vitamins, polyunsaturated fatty acids, and macro-, and trace elements. More recently, they have also revealed to be an interesting source of useful bioactive components such as antioxidants, phycocolloids, proteins, vitamins, minerals, carotenoids, soluble dietary fibers, polyunsaturated fatty acids, phycobilins, polysaccharides, sterols, tocopherols, terpenes, and phycocyanins. These compounds demonstrated to possess nutritional and functional value apart from their potential use as therapeutic agents in biomedical area (Chandini et al., 2008; Lordan et al., 2011; Mohamed et al., 2012; Alves et al., 2016b). Due to their unique structures and biochemical characteristics, the multifunctional properties of algae should be exploited in their fullness. In addition, the idea that algae are a promising prolific source of structurally unique NPs with biomedical potential is even more supported by the view that the number of algae species identified around the world is more than 30,000 (Plouguerné et al., 2014). Moreover, algae have been revealed a major source of new compounds of marine origin, after sponges, microorganisms and phytoplankton (Figure 4).

Many of these algae-derived compounds have proven therapeutic properties associated with numerous health-promoting effects, including anti-obesity (Maeda et al., 2005; Kim et al., 2018), antidiabetic (Mayer et al., 2013), antihypertensive (Sivagnanam et al., 2015), antihyperlipidaemia (Sathivel et al., 2008), antioxidant (Magalhaes et al., 2011; Pinteus et al., 2017), anticoagulant (Magalhaes et al., 2011), anti-inflammatory (De Souza et al., 2009), immunomodulatory (Pérez-Recalde et al., 2014), anti-estrogenic (Skibola, 2004), thyroid-stimulating (Teas et al., 2007), neuroprotective (Pangestuti and Kim, 2011), anti-osteoarthritic (Moon et al., 2018), anti-osteoporosis (Deng et al., 2018), antiviral (Aguilar-Briseño et al., 2015), antimicrobial (Pinteus et al., 2015; Rodrigues et al., 2015), and antitumor (Moussavou et al., 2014; Rodrigues et al., 2015; Alves et al., 2016a). Among the well-documented bioactive compounds are brominated phenols, polysaccharides, and carotenoids, but especially a large diversity of terpenoids, several of them being halogenated compounds (Gribble, 2015; Rodrigues et al., 2015). Although many works have attempted to identify marine-derived compounds, detailed chemical characterization and identification of bioactive components are still largely lacking (Santos et al., 2015).

Along the last five decades, it is estimated that more than 3,000 NPs have been discovered from algae (Leal et al., 2013), and among all of the biological activities observed, the antitumor activity is one of the most promising. Despite several studies have shown the high cytotoxic potential of the compounds isolated from algae on different tumor cell lines, there are few studies that have characterized the intracellular signaling pathways involved in the process (Table 1). Currently, according to the National Cancer Institute (USA), different targeted therapies have been approved for use in cancer treatment, including hormonal therapies, inhibitors of signal transduction and angiogenesis, modulators of gene expression, inducers of apoptosis, immunotherapies and toxin delivery molecules.

**Table 1:** Marine compounds isolated from algae with antitumor and cytotoxic activities and intracellular signaling pathways involved.

**Algae**	**Compound**	**Chemical class**	**Intracellular signaling pathways**	**References**
**OCHROPHYTA (BROWN ALGAE)**				
*Ecklonia cava*	Dieckol	Polyphenol	Induces a downregulation of FAK signaling pathway mediated by the scavenging of intracellular reactive oxygen species (ROS), influencing migration and invasion of HT1080 cells.	Park and Jeon, 2012
			Potent inhibitor for tumor promoter-mediated MAPK-signaling pathways, leading to Activator Protein 1 (AP-1) and Metalloproteinase (MMP)−9 activation by regulating cancer cell motility.	Oh et al., 2011
*Ecklonia cava*	6,6′-bieckol	Polyphenol	Acts as a suppressor of MMP-2 and MMP-9 expressions by downregulating Nuclear Factor kappa-light-chain-enhancer of Activated B cells (NF-κB) and inhibits the migration of HT1080 cells. In addition, cell morphology and shape are affected in 3D culture condition.	Zhang et al., 2010
*Ecklonia Cava*	Dioxinodehydroeckol	Phloroglucinol derivative	Induction of apoptosis through NF-κB family and NF-κB -dependent pathway.	Kong et al., 2009
*Ecklonia cava*	Fucodiphloroethol G	Phlorotannin	Promotes inhibition of AP-N, MMPs (-2,-9) and c-fos by blocking signal transduction of MAPK and Akt pathways in Vascular Endothelial Growth Factor (VEGF)-induced EVC304 and EA.hy926 cells.	Li et al., 2011
*Eisenia bicyclis*	Diphlorethohydroxycarmalol (DC)	Phlorotannin	Induces apoptosis on HL60 cells through the accumulation of sub-G1 cell population along with nuclear condensation, the reduction of Bcl-2 expression and the depletion of mitochondrial membrane potential (Δ*Ψm*).	Kang et al., 2012
*Hizikia fusiformis*	HFGP	Glycoprotein	Induces on HepG2 cells apoptosis and sub-G1 phase arrest. The expressions of Fas, Fas-associated death domain protein, Bax, and Bad were significantly upregulated in HFGP-treated cells. Moreover, HFGP induces the translocation of Bax to the mitochondria and the release of cytochrome c into the cytosol.	Ryu et al., 2012
*Hydroclathrus clathratus*	H3-a1	Sulfated polysaccharide	Induces significant arrest of sub-G1 phase on HL-60 and MCF-7 cells. *In vivo*, it inhibits tumor growth at doses of 20 and 50 mg Kg^−1^ in tumor-bearing BALB/c mice. Moreover, it suppresses ascitic sarcoma 180 tumor growth and prolongs the lifespan of the tumor-bearing mice by ~30–40%. H3-a1 compound also increases the tumor necrosis factor-alpha (TNF-α) level in mouse serum.	Wang et al., 2010
*Undaria pinnatifida*	Fucoidan	Sulfated polysaccharide	Capable of suppress the proliferation of HLF cells by AMPK-associated inhibition of fatty acid synthesis and G1/S transition.	Kawaguchi et al., 2015
			Promotes apoptosis via ROS-mediated mitochondrial pathway on SMMC-7721 cells.	Yang et al., 2013
			Induces intrinsic and extrinsic apoptosis by stimulating ERK1/2 MAPK, deactivating P38 MAPK and PI3K/Akt signaling pathways and downregulating Wnt/β-catenin signaling pathway on prostate cancer cells (PC-3).	Boo et al., 2013
*Undaria pinnatifida*	Fucoxanthin	Carotenoid	Increases the efficiency of cisplatin treatment on HepG2 cell line. Reduces cell viability. Increases Bax/Bcl-2 ratio, probably through inhibition of NF-κB, and ERCC1 expression through ERK and PI3K/AKT pathways.	Liu et al., 2013
*Cladosiphonokamuranus Tokida*	Fucoxanthinol	Carotenoid	Inhibits Akt and Activator protein-1 pathways that influenced the suppression of cell growth, migration and invasion and the induction of apoptosis on osteosarcoma cells.	Rokkaku et al., 2013
*Sargassum siliquastrum*	Sargachromanol E	Meroditerpenoid	Induction of apoptosis on HL-60 cells mediated by Caspase-3 activation. Apoptosis accompanied by downregulation of Bcl-xL, upregulation of Bax, activation of Caspase−3, and cleavage of poly (ADP-ribose) polymerase (PARP).	Heo et al., 2011
*Ascophyllum nodosum*	Ascophyllan	Sulfated polysaccharide	Reduces N-Cadherin levels and increases E-Cadherin, which lead to the inhibition of migration and adhesion of B16 cell line.	Abu et al., 2015
*Laminaria digitata*	Laminarin	Polysaccharide	Induces apoptosis and cell cycle arrest at sub-G1 and G2/M phases on human colon cancer cells (HT-29) and suppresses ErbB signaling pathway activation.	Park et al., 2013
*Laminaria japonica*	LJGP	Glycoprotein	Supresses cell proliferation and induces apoptosis on HT-29 cells mediated through Fas signaling pathway, mitochondrial pathway and cell cycle arrest.	Go et al., 2010
*Sargassum horneri*	SHPSA	Polysaccharide	Inhibits the proliferation of human colon cancer cells (DLD) by increasing the accumulation of cells at G2/M phase and inducing the apoptosis of DLD cells.	Wang S. et al., 2015
*Sargassum vulgare*	PSV1	Sulfated polysaccharide	It blocks tubulogenesis and VEGF secretion on rabbit aorta endothelial cells using Matrigel. Inhibitory effect on angiogenesis.	Guerra Dore et al., 2013
*Leathesia nana*	Bis(2,3-Dibromo-4,5- dihydroxybenzyl) ether (BDDE)	Bromophenol	Induces apoptosis on K562 cells by a mitochondrial mediated pathway. Induces ROS generation and arrests cell cycle in S phase. Interacts with the minor groove of DNA and inhibits Topoisomerase I activity.	Liu et al., 2012
			Displays *in vitro* anti-angiogenic activity by suppressing significantly vascular endothelial cells (HUVEC) proliferation, migration, and tube formation, without any effect on the preformed vascular tube. Decreases the levels of VEGF and VEGFR proteins and inhibits the VEGF downstream signaling molecules, including mTOR and Src, while activates Akt and ERK. On zebrafish embryos, blocks sub-intestinal vessel formation and exhibits toxicity when used in higher concentrations (*in vivo*).	Qi et al., 2015
*Sargassum siliquastrum*	9′-*cis*-(6′*R*) fucoxanthin (FcA) 13′-*cis*-(6′*R*) fucoxanthin complex (FcB)	Carotenoid	Both compounds reduce MMP-2, MMP-9 and mRNA levels, and the migration of HT1080 cells. Moreover, increase the expression of MMP inhibition factors (MMP-1) and suppress significantly the transcriptional activity of NF-κB, c-Jun N-terminal kinase (JNK), as well as p38 mitogen-activated protein kinase activity.	Nguyen et al., 2014
*Sargassum stenophyllum*	SargA	Sulfated polysaccharide	*In vitro*, induces a decrease of B16F10 cells migration and viability. *In vivo*, causes the inhibition of tumor growth with no systemic toxicity and exhibits an anti-angiogenic effect.	Dias et al., 2005
*Sargassum macrocarpum*	Tuberatolide B (TTB)	Meroterpenoid	TTB reduces the cell viability of several cancer cells lines (MDA-MB-231, MDA-MB-453, MCF-7, A549, H1299, HCT-116, SW620, CT26, PC-3, and DU145) by apoptosis decreasing Bcl2 expression and increasing the Caspase-3 and PARP cleavage. Promotes γH2AX foci formation and phosphorylation of several proteins (Chk2 and H2AX) related to DNA damage. In addition TTB promotes the production of ROS inhibiting STAT3 activation, which result in the decrease of the levels of cyclin D1, MMP-9, survivin, VEGF, and IL-6. Its activity seems to be mediated by ROS production and consequently inhibition of STAT3 signaling.	Choi et al., 2017
*Stoechospermum marginatum*	5(*R*), 19-diacetoxy-15,18 (*R* and *S*), dihydro spata-13, 16(*E*)-diene (DDSD)	Spatane diterpenoid	*In vitro* DDSD induces cell cycle arrest at the S-phase and cell death by apoptosis on B16F10 melanoma cells. This compound promotes the generation of ROS, and consequently alterations in the ratio of Bax/Bcl-2 and in the mitochondrial transmembrane potential (ΔΨm), phosphatidylserine externalization, release of cytochrome c to the cytoplasm, Caspase activation, nuclear condensation, and fragmentation of DNA. Moreover, the results suggest that DDSD induces apoptosis through deregulating PI3K/AKT signaling pathway. *In vivo*, DDSD inhibits tumor growth (volume and weight) without evident toxic effects on C57BL/6 mice bearing B16F10 melanoma.	Velatooru et al., 2016
Not identified by the authors	MSP	Sulfated polysaccharide	Exhibits anti-metastatic ability, both *in vitro* and *in vivo*. Induces regulatory effects on Actin dynamics in an FAK/ERK1/2-dependent manner, which might be further attributed to its binding to FN and, consequently, FN-induced tumor adhesion, and migration.	Tang et al., 2006
Not identified by the authors	Not defined	Sulfated polysaccharide	Induces apoptosis and cell arrest at G2/M phase of MKN45 cells via ROS/JNK signaling pathway. In addition, it promotes ROS production and mediate the phosphorylation of several proteins, including Jun N-terminal kinase (JNK), p53, Caspase-9, and -3.	Xie et al., 2016
**RHODOPHYTA (RED ALGAE)**				
*Lophocladia sp*.	Lophocladines B	Alkaloid	Cell cycle analysis on MDA-MB-435 cells showed arrest at G2/M phase and induction of microtubule depolymerization on A-10 cells.	Gross et al., 2006
*Laurencia viridis*	Polyether triterpenoid dehydrothyrsiferol	Terpenoid	Induces apoptosis on breast cancer cells by estrogen-depend and independent pathways.	Pec et al., 2003
*Eucheuma serra*	*Eucheuma serra* agglutinin (ESA)	Lectin	Increases Caspase-3 expression and translocation of phosphatidylserine in lectin-treated colon26 cells, suggesting that cell death is mediated by apoptosis. *In vivo* is observed a significant growth inhibition of Colon26-induced tumors on BALB/c mice. DNA fragmentation in tumor cells after intravenous injection with ESA is also detected.	Fukuda et al., 2006
*Gracilaria verrucosa*	(*E*)-9- oxooctadec-10-enoic acid (C10)	Enone fatty acid	Angiogenesis and NF-κB activation in HUVECs cells stimulated by VEGF are blocked as well as their proliferation and migration. This is also observed *in vivo* model of angiogenesis using mouse cornea. Moreover, the neovascularization induced by VEGF is significantly suppressed.	Furuno et al., 2011
*Grateloupia filicina*	GFP08	Sulfated polysaccharide	In the chicken chorioallantoic membrane assay, reduced new vessel formation. In mice decreases the weight of sarcoma-180 cells-induced tumor in a dose-dependent manner. Also decreased Tissue Factor (TF) expression without affecting the activities of MMP-2 and−9.	Yu et al., 2012
*Laurencia intricata*	Laurenditerpenol	Diterpene	Inhibits hypoxia-inducible factor-1 (HIF-1) mediated hypoxic signaling in breast tumor cells.	Mohammed et al., 2004
*Laurencia papillosa*	Sulfated carrageenan (ESC)	Sulfated polysaccharide	Inhibits MDA-MB-231 cell proliferation and induces cell death through nuclear condensation and DNA fragmentation. Cell death is induced by apoptosis as result of activation of the extrinsic apoptotic Caspase-8 gene. The apoptotic signaling pathway is regulated through the Caspase-3, Caspase-9, p53, Bax, and Bcl-2 proteins.	Murad et al., 2015
*Laurencia majuscula*	Hexadecyl-1- *O*-α-l-arabinopyranoside	Arabinopyranoside	Decreases significantly CDK1 and Cyclin A expression, with slight changes in Cyclin B1; arrests cell cycle at G2/M.	Du et al., 2010
*Callophycus serratus*	Bromophycolide A	Diterpene–benzoate macrolides	Induces apoptosis on A2780 human ovarian cells; arrests G1 phase of the cell cycle, consistent with decreased number of cells from the S and G2/M phases.	Kubanek et al., 2005
*Chondrus ocellatus*	λ-Carrageenan	Sulfated galactan	Conjugation with 5-Fluorouracil (5-FU) enhanced antitumor activity and mitigated immunocompetence damage of 5-FU.	Zhou et al., 2006
*Laurencia microcladia*	Elatol	Sesquiterpene	Induces cell cycle arrest at G1 and sub-G1 phases, leading cells to undergo apoptosis. Reduces the expressions of Cyclin-D1, Cyclin-E, Cyclin-dependent kinase (Cdk)2 and Cdk4. It is also observed increases in Bak, Caspase-9 and p53 expressions and a decrease in Bcl-xl expression. *In vivo* elatol treatment reduces tumor growth on C57Bl6 mice.	Campos et al., 2012
*Laurencia thyrsifera*	Thyrsiferol	Triterpene	Supresses HIF-1 activation on T47D human breast tumor cells and blocks mitochondrial respiration at complex I.	Mahdi et al., 2011
*Champia feldmannii*	Cf-PLS	Sulfated polysaccharide	*In vivo* antitumor activity without marked toxicity. Enhances the efficacy of 5-FU, while preventing immunocompetence hindrance by 5-FU.	Lins et al., 2009
*Porphyra haitanensis*	Porphyran	Sulfated galactan	Conjugation with 5-FU enhanced its antitumor activity and mitigated immunocompetence damage.	Wang and Zhang, 2014
*Porphyra yezoensis*	PY-D2	Polysaccharide	Blocks cell cycle at G0/G1 or G2/M check-points on different cell lines (SMMC-7721, HO-8910, MCF-7, K562 cells).	Zhang et al., 2011
*Porphyra yezoensis*	Sulfoquinovosyldiacylglycerol (SQDG)	Sulfolipids	Inhibits significantly telomerase activity.	Eitsuka et al., 2004
*Grateloupia elliptica*	Pheophorbide a (Pa)	Chlorophyll	Induces cytostatic activity on glioblastoma cells (U87 MG). The cell cycle distribution showed that U87 MG cells are arrested at G0/G1 phase.	Nguyen et al., 2014
*Grateloupia longifolia*	GLP	Polysaccharide	Prevents the proliferation of HMEC-1 and HUVEC cells, suppresses the formation of intact tube networks and decreases migration. Decreases vessels density and new vessels formation in the chick chorioallantoic membrane assay and also, by intravenous administration decreases tumor weight and vascular density without showing toxicity in mice bearing sarcoma-180-cells-induced tumors.	Zhang et al., 2006
*Rhodomelaceae confervoides*	Bis-(2,3-dibromo-4,5-dihydroxy-phenyl)-methane (BDDPM)	Bromophenol	Inhibits several biological processes associated with angiogenesis, including endothelial cell sprouting, migration, proliferation, and tube formation.	Wang B. et al., 2015
*Symphyacladia latiuscula*	2,3,6-tribromo-4,5-dihydroxybenzyl methyl ether (TDB)	Bromophenol	Inhibits MCF-7 breast cancer cells growth and induces DNA fragmentation by apoptosis, accompanied by a downregulation of Bcl-2 protein expression and PARP cleavage by Caspase-3. This treatment increases the level of p21 WAF1/CIP1 protein in a p53-dependent manner.	Lee et al., 2007
*Pterocladiella capillacea*	Mertensene	Halogenated monoterpene	Induces apoptosis on HT-29 cells accompanied by Caspase-3 activation and PARP cleavage. Decreases the phosphorylated forms of several proteins (p53, Rb, Ccd2, Chkp2) and the levels of cyclin-dependent kinases CDK2 and CDK4, and increases the levels of death receptor-associated protein TRADD. In addition it seems to promote the activation of MAPK ERK-1/-2, Akt and NF-κB pathways.	Tarhouni-Jabberi et al., 2017
**CHLOROPHYTA (GREEN ALGAE)**				
*Avrainvillea nigricans*	Nigricanosides A (NA)	Glycolipid	Arrests MCF-7 breast cancer cells in mitosis. Cells exhibit disorganized microtubule spindles. *In vitro* induces polymerization of Tubulin and inhibition of both MCF-7 and HCT-116 cells proliferation.	Williams et al., 2007
*Caulerpa* spp.	Caulerpin	Alkaloid	Acts as an inhibitor of the transportation of electrons to mitochondrial complex III, interfering with the mitochondrial ROS-regulated HIF-1 activation and HIF-1 downstream target genes expression.	Liu et al., 2009
*Caulerpa taxifolia*	Caulerpenyne	Sesquiterpenoid	An early shift into synthesis phase (S) along with a blockade at G2/M phase is observed on colorectal cancer cells.	Fischel et al., 1994
*Codium fragile*	Siphonaxanthin	Carotenoid	Induces apoptosis on HeLa cells accompanied by a decrease of Bcl-2 expression and subsequently activation of Caspase-3 and increase of the expression of GADD45α and the Death Receptor 5 (DR5).	Ganesan et al., 2011
*Ulva intestinalis*	EI-SP	Sulfated polysaccharide	Induces apoptosis on HepG2 cells accompanied by changes in mitochondrial membrane potential, release of cytochrome c to the cytosol, decrease and increase of Bcl-2 and Bax expression, respectively and cleavage of Caspase-3 and Caspase-9, as well as cleavage of PARP.	Wang et al., 2014
*Ulva intestinalis*	DAEB	Sulfated polysaccharide	Exhibits low toxicity *in vitro. In vivo* DAEB reduces tumor mass and increases thymus and spleen mass. Tumor growth inhibition is ascribed to increase levels of TNF- α, NO, and ROS.	Jiao et al., 2009
*Capsosiphon fulvescens*	Cf-GP	Glycoprotein	Inhibits AGS cells proliferation and migration by a decrease of Integrin expression via the TGF-β 1-activated FAK/PI3K/AKT pathways.	Boo et al., 2013
*Capsosiphon fulvescens*	Cf-PS	Polysaccharide	Inhibits cell proliferation and induces apoptosis by inhibiting IGF-IR signaling and the PI3K/Akt pathway.	Kwon and Nam, 2007
*Codium fragile*	Clerosterol	Sterol	Induces apoptosis accompanied by changes in mitochondrial membrane potential, an increase and a decrease of Bax and Bcl-2 expression, respectively, and activation of Caspase-3 and Caspase-9.	Kim et al., 2013
*Codium decorticatum*	GLP	Glycoprotein	GLP induces apoptosis on MDA-MB-231 breast cancer cells by mitochondria-mediated intrinsic pathway promoting changes in the mitochondrial membrane potential and Bax/Bcl-2 ratio, cytochrome c release, and Caspases-3 and 9 activation.	Thangam et al., 2014

### *In vitro* antitumor activities of algae-derived compounds and intracellular signaling pathways activated

Analyzing the compounds isolated from algae with antitumor activity (Table 1) is possible to see that several of them mediate their activities in some of these target therapies mentioned previously. For example, dioxinodehydroeckol (Kong et al., 2009), sargachromanol E (Heo et al., 2011), EI-SP (Wang et al., 2014), siphonaxanthin (Ganesan et al., 2011), sulfated carrageenan (Murad et al., 2015), TDB (Lee et al., 2007), GLP (Thangam et al., 2014), mertensene (Tarhouni-Jabberi et al., 2017), TTB (Choi et al., 2017), DDSD (Velatooru et al., 2016), and clerosterol (Kim et al., 2013) induced apoptosis in different cell lines by similar intracellular signaling pathways, regulated by Caspase (−3, −9 or both) activation, downregulation of Bcl-xL or Bcl-2, upregulation of Bax and cleavage of PARP. Moreover, some of these compounds, such as EI-SP and clerosterol, also caused the loss of the mitochondrial membrane potential. The treatment of colon26 cells with *Eucheuma serra* agglutinin (Fukuda et al., 2006) also promoted an increase of Caspase-3 expression and translocation of phosphatidylserine in lectin-treated cells, suggesting that cell death was mediated by apoptosis. On the other hand, algae-derived compounds such as lophocladines B (Gross et al., 2006), hexadecyl-1- *O*-α-l-arabinopyranoside (Du et al., 2010) and caulerpenyne (Fischel et al., 1994) affected the intracellular signaling pathways linked with regulation of the cell cycle. Lophocladines B showed a marked reduction of MDA-MB-435 cells at the G1 and S phases, with an accumulation of cells at G2/M, indicating a G2/M cell cycle arrest. This compound also induced microtubule depolymerization on A-10 cells. Du et al. (2010) isolated from the alga *Laurencia majuscula* a new arabinopyranoside compound designed as hexadecyl-1- *O*-α-l-arabinopyranoside, which exhibited significant antitumor activity in different cancer cell lines. The active compound arrested cell lines at G2/M phase of the cell cycle by decreasing the expression of CDK1 and Cyclin A proteins, which are critical for the G2/M-phase transition. Caulerpenyne induced cell cycle arrest in colorectal cancer cells that exhibited an early shift into the S phase followed by a blockade at the G2/M phase (Fischel et al., 1994).

Nevertheless, most of the intracellular signaling pathways activated by these compounds are simultaneously linked with the regulation of cell cycle and apoptosis. Park et al. (2013) demonstrated that laminarin, extracted from brown alga *Laminaria digita*, induced apoptosis on HT-29 colon cancer cells and increased the percentage of cells in the sub-G1 and G2/M phases. The observed decrease in cellular proliferation was found to be dependent on ErbB, followed by subsequent activation of c-Jun N-terminal kinase. In the same way, Liu et al. (2012) verified that a bromophenol compound, bis(2,3-dibromo-4,5- dihydroxybenzyl) ether, induced apoptosis on K562 cells by a mitochondria-mediated pathway, as well as the arrest of the cell cycle at the S phase. Additionally, this compound interacted with the minor groove of DNA and inhibited Topoisomerase I activity. On A2780 human ovarian cells, bromophycolide A induced the arrest at the G1 phase of the cell cycle and a consequent and consistent loss of cells from the S and G2/M phases, while simultaneously induced apoptosis (Kubanek et al., 2005). Similar effects were induced by elatol, a compound isolated from the alga *Laurencia microcladia*, which induced cell cycle arrest in the G1 and sub-G1 phases, leading the cells to undergo apoptosis. It influenced the expression of several proteins (cyclins, Bax, Bcl-xl, caspases, p53) that play important roles in these biological processes (Campos et al., 2012). Diphlorethohydroxycarmalol, isolated by Kang et al. (2012), induced apoptosis by the accumulation of the sub-G1 cell population and nuclear condensation, depletion of mitochondrial membrane potential (ΔΨm) and regulation of the expression of the pro-survival and pro-apoptotic Bcl-2 family members. HFGP (Ryu et al., 2012) and LJGP (Go et al., 2010) glycoproteins induced apoptosis on HepG2 and HT-29 cells, which was mediated by Fas signaling and mitochondrial pathway, and cell cycle arrest. On the other hand, Cf-PS polysaccharide (Kwon and Nam, 2007) inhibited the cell proliferation and induced apoptosis by inhibiting IGF-IR signaling and the PI3K/Akt pathway, which are involved in the regulation of cell growth, proliferation, differentiation, motility, survival, metabolism and protein synthesis (Chen et al., 2014).

Other examples such as dieckol (Oh et al., 2011; Park and Jeon, 2012), 6,6′-bieckol (Zhang et al., 2010), ascophyllan (Abu et al., 2015), 9′-*cis*-(6′*R*) fucoxanthin (FcA) (Nguyen et al., 2014), fucoxanthinol (Rokkaku et al., 2013), and 13′-*cis*-(6′*R*) fucoxanthin complex (FcB) (Nguyen et al., 2014), SargA (Dias et al., 2005), MSP (Tang et al., 2006), and Cf-GP (Boo et al., 2013) showed the capacity to inhibit the motility, migration, adhesion or invasion on different *in vitro* and *in vivo* models using distinct intracellular signaling pathways, as described in Table 1.

Currently, one of the targets of cancer treatment, especially in solid tumors, is angiogenesis, which is responsible for the formation of new blood vessels and is a requirement for the sustained growth and proliferation of solid tumors. Accordingly, the search for inhibitors of this process has become a leading line of investigation in anticancer research, with the consequent release of several drugs on the market that have clearly improved outcomes in patients with different tumor types and metastatic disease (Marín-Ramos et al., 2015). The compounds PSV1 (Guerra Dore et al., 2013), SargA (Dias et al., 2005), BDDE (Qi et al., 2015), GFP08 (Yu et al., 2012), GLP (Zhang et al., 2006), Fucodiphloroethol G (Li et al., 2011), C10 (Furuno et al., 2011), and BDDPM (Wang B. et al., 2015) also demonstrated interesting anti-angiogenic activities. For example, the sulfated polysaccharide, PSV1, inhibited tubulogenesis in RAEC cells in Matrigel and VEGF secretion (Guerra Dore et al., 2013); SargA induced a marked dose-dependent inhibition of capillary networks development (Dias et al., 2005). As to fucodiphloroethol G, this compound inhibit angiogenesis on ECV-304 and EA.hy926 cells when induced with VEGF, as well as the transcriptional factor c-fos and its targets AP-N, MMP-2, by MAPK, and Akt signaling pathways inhibition (Li et al., 2011). However, one of the most interesting and promising compounds is BDDPM, reported to inhibit various biological processes associated with angiogenesis, including endothelial cell sprouting, migration, proliferation, and tube formation (Wang B. et al., 2015). Kinase assays revealed that BDDPM is a potent selective but multi-target receptor tyrosine kinase (RTKs) inhibitor (VEGFR, PDGFR, FGFR, and EGFR). However, other compounds have not only evidenced *in vitro* activity but also *in vivo* as described in the section Preclinical and Clinical Evidence of Antitumor Activities of Algae-Derived Compounds of the present review.

Compounds such as laurenditerpenol (Mohammed et al., 2004), caulerpin (Liu et al., 2009), thyrsiferol (Mahdi et al., 2011), phlorofucofuroeckol-A (Lee et al., 2012), SQDG (Eitsuka et al., 2004), and DAEB (Jiao et al., 2009) showed antitumor activity by activating other intracellular signaling pathways. For example, laurenditerpenol, thyrsiferol, and caulerpin showed the capacity to inhibit the transcription factor HIF-1 by blocking the induction of the oxygen-regulated HIF-1α protein, which promotes tumor cell adaptation and survival under hypoxic conditions (Ke and Costa, 2006). Lee et al. (2012) observed that phlorofucofuroeckol-A, a compound isolated from the edible brown alga *Eisenia bicyclis*, is a potent inhibitor of the aldo-keto reductase family 1 B10 (AKR1B10), a member of the NADPH-dependent aldo-keto reductase (AKR) superfamily, considered to be a potential cancer therapeutic target. In the same way, the compound SQDG showed a marked inhibition of the telomerase activity, which is an enzyme that drives the uncontrolled division and replication of cancer cells (Eitsuka et al., 2004).

Some of the compounds isolated from algae can also be used as co-adjuvants to improve the efficiency of the drugs currently used as therapeutics. For instance, the pre-treatment of HepG2 cells with fucoxanthin allowed to improve the therapeutic effect of cisplatin (Yang et al., 2013). According to Liu et al. (2013), these effects were associated with NFκB expression inhibition and an increase in the Bax/Bcl-2 mRNA ratios regulated by NFκB. Moreover, the decrease of the DNA repair systems regulated by ERK, p38, and PI3K/AKT seems also be associated with these effects. Additionally, the conjugation of λ-carrageenan (Zhou et al., 2006), Cf-PLS (Lins et al., 2009), and porphyran (Wang and Zhang, 2014) compounds with 5-FU drug enhanced its antitumor activity. According to previous studies, the conjugation of these compounds with 5-FU increased the antitumor activities of the drug and mitigated the immunocompetence damage induced by 5-FU (Zhou et al., 2006; Lins et al., 2009; Wang and Zhang, 2014).

**Figure 5:**
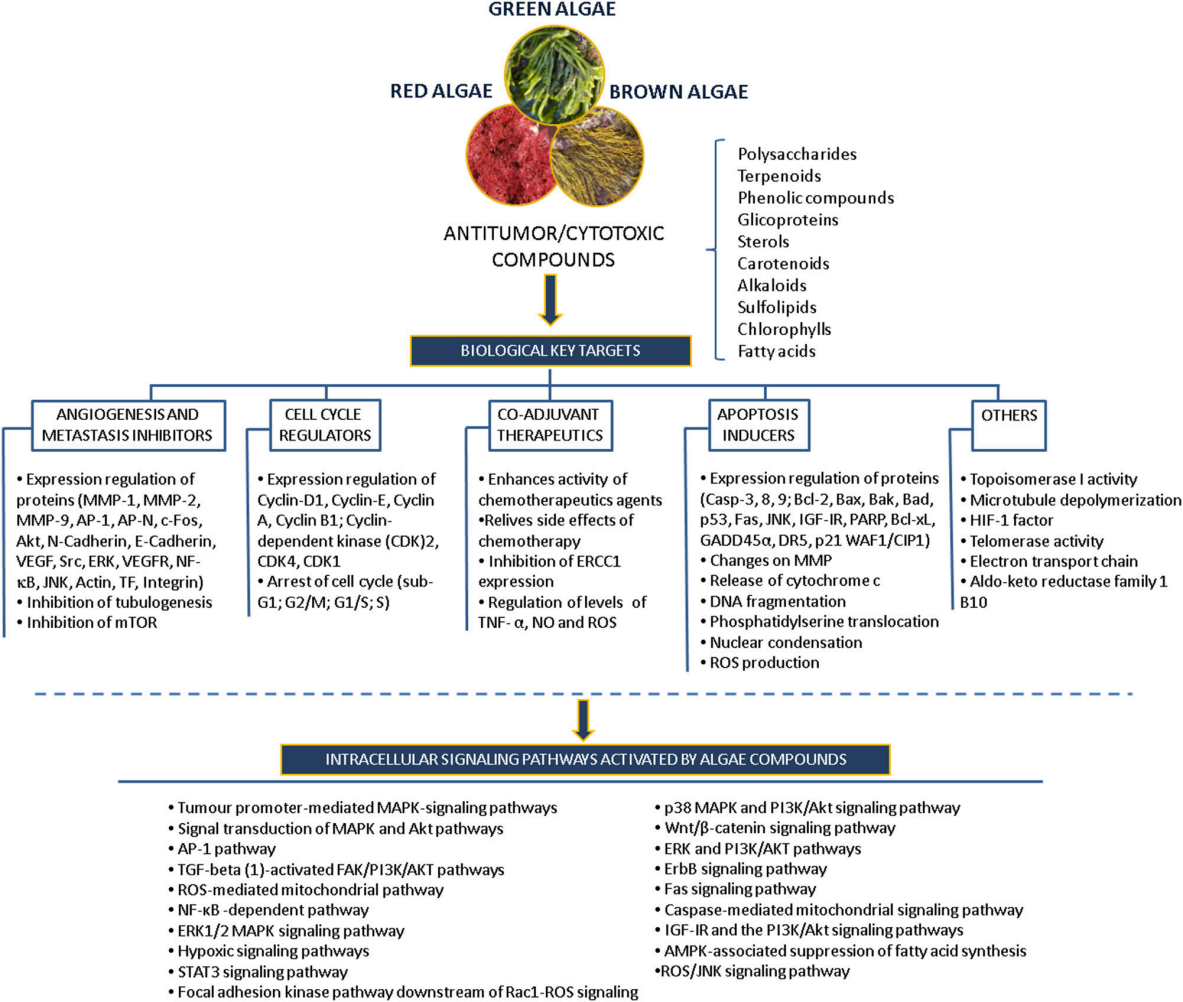
Overview of the antitumor/cytotoxic compounds isolated from algae, biological targets and intracellular signaling pathways activated.

Many of the studies discussed above have identified compounds, such as polysaccharides, polyphenols, carotenoids, alkaloids, terpenes and others, that mediate specific inhibitory activity on a number of key cellular processes, including apoptosis pathways, angiogenesis, migration and invasion processes, in different *in vitro* models revealing their potential use as anticancer drugs (Figure 5).

### Preclinical and clinical evidence of antitumor activities of algae-derived compounds

Despite the antitumor activities of algae-derived compounds have majorly been described on *in vitro* human tumor models, there are several studies in preclinical and clinical trials demonstrating the potential of these compounds as antitumor and/or co-adjuvants drugs with capacity to act in distinct intracellular signaling pathways.

Among the chemical structures derived from algae, the highest number of *in vivo* studies were conducted with sulfated polysaccharides, revealing their potential to be used in antitumor therapies. For instance, the sulfated polysaccharides H3-a1 (Wang et al., 2010) and DAEB (Jiao et al., 2009) revealed antitumor activity by immune system enhancement, through the increase of the tumor necrosis factor-alpha (TNF-α) and ROS levels and by the activation of peritoneal macrophages leading to the secretion of TNF-α and NO. In addition, when administrated in tumor-bearing BALB/c mice at doses of 20 and 50 mg/Kg, the sulfated polysaccharide H3-a1 exhibited capacity to suppress the ascitic sarcoma 180 tumor growth, increasing the lifespan of the tumor-bearing mice in ~30–40%. On the other hand, the treatment with the sulfated polysaccharide DAEB reduced the thymus and spleen tumor growth in mice promoting the increase of immune organs weight.

Due to increasing evidences of the antitumor potential of algae derived sulfated polysaccharides, several studies have been conducted to deepen their antitumor potential, including studies addressing the possibility to be used as co-adjuvants drugs, to increase treatments efficiency and also as an attempt to reduce undesirable side-effects. As an example, the sulfated polyssacharide Cf-PLS extracted from the red alga *C. feldmannii* exhibited capacity to reduce the tumor growth on mice transplanted with sarcoma 180 tumor cells and to increase the leukocytes number in the peritoneal cavity and neutrophil migration. In addition, its co-administration with the chemotherapeutic agent 5-FU on tumor-bearing animals allowed to significantly increase the tumor growth inhibition, comparing with single administration, also preventing the immunocompetence hindered by 5-FU (Lins et al., 2009).

As described above, one of the targets for cancer treatment is angiogenesis. In these studies, several compounds have exhibited promising activities on *in vivo* models. The treatment of mice bearing sarcoma-180 cells with GLP polysaccharide promoted the reduction of tumor weight around 52% and the vascular density of the tumor (Zhang et al., 2006). In the same model, the GFP08 compound decreased the tumor weight by 68.9% and inhibited the formation of new vessels in the chicken chorioallantoic membrane assay (*ex vivo*) (Yu et al., 2012). By other side, the bromophenol BDDE also exhibited anti-angiogenesis properties by inhibiting sub-intestinal vessel formation in zebrafish embryos *in vivo* (Qi et al., 2015). Similarly to the bromophenol BDDE, the compound C10 inhibited markedly the neovascularization induced by VEGF in the mouse cornea (Furuno et al., 2011). On the other hand, the compound SargA inhibited the vascularization of Gelfoam skin implants and exhibited antitumor properties on melanoma cells with doses of 1.5 and 150 μg/animal without inducing deaths or body weight loss (Dias et al., 2005).

The MSP sulfated polysaccharide mediated a potent inhibitory effect on the metastasis of Lewis lung carcinoma reducing significantly the number of pulmonary metastatic colonies. The reduction of colony-formation rate was between 70 and 93.6%. Moreover, compared with cisplatin treatment, this compound promoted a significant amelioration of alveolar structures without inducing weight loss (Tang et al., 2006). DDSD (4, 10, and 15 mg/Kg) and Elatol [(oral (3, 10, 30 mg/Kg) or intraperitoneal (1, 3, 10 mg/Kg)] demonstrated significantly reduction of the tumor growth without evident toxic effects (Campos et al., 2012; Velatooru et al., 2016). The lectin ESA extracted from red alga *Eucheuma serra*, injected in the tail vein of BALB/c mice with colon26 cells, promoted a significant delay of the tumors growth inducing the cells death by apoptosis as observed *in vitro*. Additionally the treatment with ESA compound did not promoted weight loss or animal death (Fukuda et al., 2006). These studies become more relevant since the antitumor activities mediated by many of these compounds are not linked with toxic effects.

Other of the compounds widely studied on *in vivo* models, including clinical trials, is the sulfated polyssacharide fucoidan. This compound exhibits multi-targets acting in different signaling pathways, including the activation of the intrinsic and extrinsic pathways of apoptosis, suppression of angiogenesis, increase immune response, and mobilization of haematopoietic progenitor cells (Kwak, 2014; Moghadamtousi et al., 2014). The co-administration of low-molecular –weight fucoidan with standard drugs in patients with metastatic colorectal cancer improved the disease control rate suggesting its potential application as additional therapy (Tsai et al., 2017). In addition when administrated in conjugation with the standard hormonal drugs letrozole and tamoxifen, in patients with breast cancer, revealed to be well tolerated and without influence in the steady-state plasma concentrations of these drugs (Tocaciu et al., 2018). Despite de great potential demonstrated by fucoidans on clinical trials, to be used as suplementary therapy, its use as anticancer drugs is being studied, since fucoidan preparations obtained from different sources have induced different anticancer activities *in vivo*. These differential responses seem to be associated with their different structural properties. Therefore, it will be important to determine the structural characteristics of fucoidan responsible for the verified *in vivo* antitumor activities to ensure its potential use as therapeutic agent (Kwak, 2014).

The marine-derived cyclic depsipeptide kahalalide F was the first compound found in algae that achieved the phase II of clinical trials (Murphy et al., 2014). Kahalalide F is a potent cytotoxic compound produced by the green alga *Bryopsis pennata* and found in the mollusk *Elysia rufescens* (Miguel-Lillo et al., 2015; Sable et al., 2017). It advanced through five clinical trials and completed the safety evaluation in phase I in patients with distinct advanced solid tumors. Nevertheless, kahalalide F dropped in phase II due to lack of efficacy, short half-life, limited spectrum of activity and a poor response in patients. However, due the high potential of this compound as cytotoxic, it inspired the development of several synthetic analogs to overcome its limitations increasing its potency and half-life time (Wang B. et al., 2015).

In the last years nanotechnology has emerged as a promising solution to be used in drug delivery systems to suppress some of these limitations, being considered as one of the next-generation platform for cancer therapy (Sun et al., 2014; Xin et al., 2017). Therefore the production of nano-formulations of drugs derived from algae-derived compounds can be an interesting approach to potentiate anticancer properties. In addition the use of nano-formulations can also be useful to overcome some limitations that arise from specific characteristics of each compound, such as nonspecific biodistribution, low water solubility, lack of targeting capability, systemic toxicity, weak therapeutic effect, and limited bioavailability (Sun et al., 2014; Blanco et al., 2015). For instance one of the most promising compounds isolated from algae with interesting antitumor proprieties is fucoxanthin. However, the poor solubility, chemical instability, and low bioavailability of this carotenoid limit its use in cancer therapeutics. To overcome these drawbacks several approaches have been assessed such as the inclusion of fucoxanthin in nano-emulsions (Huang et al., 2017), nano-suspensions (Muthuirulappan and Francis, 2013) and nano-gels (Ravi and Baskaran, 2015). However as described by Bajpai et al. (2018) for the microalgae, the published data on nano-formulations using compounds derived from macroalgae is also scarce suggesting a new area to be explored that can potentiate the capacity of these compounds in cancer therapeutics.

According to the interesting activities mediated by algae-derived compounds, it is expected that in the next few years some of them may reach the clinical trials stages or inspire the development of new compounds allowing their translation into clinically useful drugs in the future. Moreover, the use of some those compounds as co-adjuvant in pre-existent therapeutics regimes appears to be a valid approach to improve the therapeutic effects of the antitumor drugs and decrease their side-effects.

### The therapeutic potential of *Sphaerococcus coronopifolius* compounds—case study

*Sphaerococcus coronopifolius* is a red alga belonging to the Rhodophyta phylum, which is narrow, compressed, two-edged, cartilaginous, scarlet fronds and main axes that are dark brownish-red. The habitat of this species is rarely on rocks in the lower littoral, but it is common in the shallow sublittoral to a 15 m depth. They are distributed in the East Atlantic (Ireland and Britain to Canary Islands) and Mediterranean and Black Seas (Guiry and Guiry, 2015). Since its first chemical analysis in 1976, *S. coronopifolius* has demonstrated to be an interesting source of brominated cyclic diterpenes, most of them containing one or two bromine atoms (Rodrigues et al., 2015). Although more than 40 compounds have been isolated and described from *S. coronopifolius*, in the last four decades, few studies characterized their biological activities. According to previous studies, some of the compounds isolated evidenced great biological activities, including antifouling (Piazza et al., 2011), antimalarial (Etahiri et al., 2001) and antimicrobial (Etahiri et al., 2001; Smyrniotopoulos et al., 2010b,c; Rodrigues et al., 2015). Another interesting bioactivity exhibited by these compounds is their antitumor potential. Several compounds revealed interesting cytotoxic activities in different *in vitro* models (Table 2). For example, 14*R*-Hydroxy-13,14-dihydro-sphaerococcenol-A decreased significantly the viability of NSCLC-N6-L16 and A549 human lung cancer cell lines with an IC_50_ of 5 and 4 μg/mL, respectively. Moreover, spirosphaerol and corfusphaeroxide showed moderate cytotoxic activity against the malignant cell lines A549, Hs683 and MCF-7. Smyrniotopoulos et al. (2010a) isolated several metabolites and evaluated their cytotoxic activity toward four human apoptosis-resistant (U373, A549, SK-MEL-28, OE21) and two human apoptosis-sensitive (PC-3, LoVo) cancer cell lines with IC_50_ values ranging from 3 to 100 μM. In a study performed by Rodrigues et al. (2015), the compound Sphaerococcenol A exhibited the highest anti-proliferative activity on HepG2 cells with an IC_50_ of 42.87 μM, being more potent than cisplatin (75.41 μM). Although some compounds isolated from *Sphaerococcus coronopifolius* have exhibited interesting biological activities, its potential remains understudied since the intracellular mechanisms associated with the observed effects are yet uncovered. *S. coronopifolius* is therefore a clearly example of the potential of algae as source of cytotoxic compounds, still under explored, suggesting that the potential of algae as source of compounds for the development of new antitumor drugs is probably much higher than previously thought.

**Table 2:** Cytotoxic compounds isolated from *Sphaerococcus coronopifolius*.

**Compound name**	**First report**	**Locale of collection**	**Chemical structure**	**Cytotoxic activities (IC_50_ in μM or μg/mL)**	**References**
Sphaerococcenol A	1976	La Escala, Spain	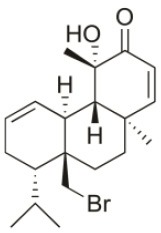	U373 (IC_50_:3.2 μM)^*^; A549 (IC_50_:3.7 μM); SK-MEL-28 (IC_50_:5.2 μM); OE21 (IC_50_:3 μM); PC-3 (IC_50_:3.7 μM); LoVo (IC_50_:2.8 μM); HepG2 (IC_50_:42.87 μM)	Fenical et al., 1976; Smyrniotopoulos et al., 2010a; Rodrigues et al., 2015
Bromosphaerol	1976	Italy	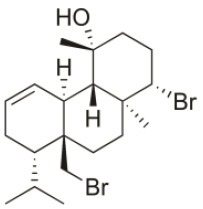	U373 (IC_50_:30 μM); A549 (IC_50_:35 μM); SK-MEL-28 (IC_50_:34 μM); OE21 (IC_50_:28 μM); PC-3 (IC_50_:30 μM); LoVo (IC_50_:23 μM); HepG2 (IC_50_:203.33 μM)	Fattorusso et al., 1976; Smyrniotopoulos et al., 2010a; Rodrigues et al., 2015
Bromosphaerodiol	1977	Portopalo, Sicily, Italy	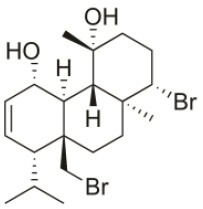	U373 (IC_50_:22 μM); A549 (IC_50_:24 μM); SK-MEL-28 (IC_50_:31 μM); OE21 (IC_50_:15 μM); PC-3 (IC_50_:26 μM); LoVo (IC_50_:20 μM)	Smyrniotopoulos et al., 2010a
12*S*-Hydroxy-bromosphaerol	1982	Bay of Salerno, Italy	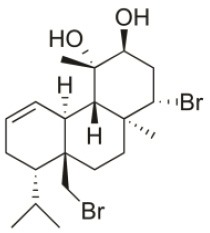	HepG2 (IC_50_:291.42 μM); U373 (IC_50_:16 μM); A549 (IC_50_:19 μM), SK-MEL-28 (IC_50_:22 μM); OE21 (IC_50_:19 μM); PC-3 (IC_50_:12 μM); LoVo (IC_50_:9 μM)	Cafieri et al., 1982; Smyrniotopoulos et al., 2010a; Rodrigues et al., 2015
1*S*-Hydroxy-1,2-dihydro-bromosphaerol	1982	Bay of Salerno, Italy	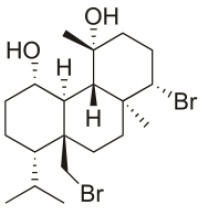	U373 (IC_50_:25 μM); A549 (IC_50_:28.6 μM); OE21 (IC_50_:20 μM); SK-MEL-28 (IC_50_:26 μM); PC-3 (IC_50_: 25 μM); LoVo (IC_50_: 23 μM)	Cafieri et al., 1982; Smyrniotopoulos et al., 2010a
Bromotetrasphaerol	1986	Bay of Napoles, Massalubrense, Italy	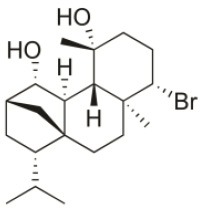	U373 (IC_50_:34 μM); A549 (IC_50_:38 μM); OE21 (IC_50_:33 μM); SK-MEL-28 (IC_50_:43 μM); PC-3 (IC_50_:43 μM); LoVo (IC_50_:56 μM)	Cafieri et al., 1986; Smyrniotopoulos et al., 2010a
12*R*-Hydroxy-bromosphaerol	1987	Bay of Naples, Massalubrense, Italy	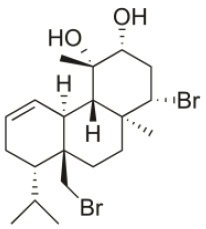	HepG2 (IC_50_:104.83 μM); U373 (IC_50_:25 μM); A549 (IC_50_:28 μM); OE21 (IC_50_:25 μM); SK-MEL-28 (IC_50_:29); PC-3 (IC_50_: 26 μM); LoVo (IC_50_: 26 μM)	Cafieri et al., 1987; Smyrniotopoulos et al., 2010a; Rodrigues et al., 2015
Alloaromadendrene	1988	Plomin, Croatia	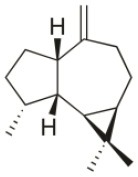	U373 (IC_50_:71 μM); A549 (IC_50_:79 μM); OE21 (IC_50_:83 μM); PC-3 (IC_50_: 35 μM); LoVo (IC_50_: 63 μM)	de Rosa et al., 1988; Smyrniotopoulos et al., 2010a
1*S*-Hydroperoxy- 12*R*-hydroxy-bromosphaerol-B	2008	Palaiokastritsa bay, Corfu Island, Greece	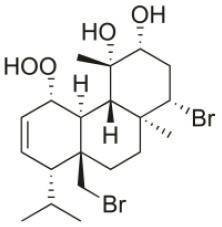	NSCLC-N6-L16 (IC_50_: 9.5 μg/mL); A549 (IC_50_:12 μg/mL); U373 (IC_50_:32 μM); A549 (IC_50_:40 μM); OE21 (IC_50_:25 μM); SK-MEL-28 (IC_50_:31 μM); PC-3 (IC_50_: 30 μM); LoVo (IC_50_: 22 μM)	Smyrniotopoulos et al., 2008, 2010a
1*S*-Hydroperoxy-12*S*-hydroxy-bromosphaerol-B	2008	Palaiokastritsa bay, Corfu Island, Greece	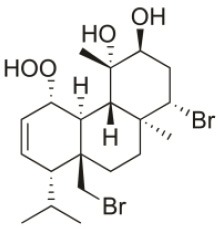	NSCLC-N6-L16 (IC_50_: 6 μg/mL); A549 (IC_50_:5 μg/mL); U373 (IC_50_:22 μM); A549 (IC_50_:26 μM); OE21 (IC_50_:27 μM); SK-MEL-28 (IC_50_:28 μM); PC-3 (IC_50_: 28 μM); LoVo (IC_50_: 28 μM)	Smyrniotopoulos et al., 2008, 2010a
14*R*-Hydroxy-13,14-dihydro-sphaerococcenol-A	2008	Palaiokastritsa bay, Corfu Island, Greece	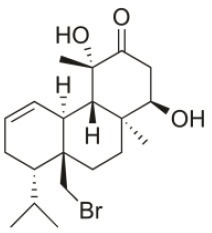	NSCLC-N6-L16 (IC_50_:5 μg/mL); A549 (IC_50_: 4 μg/mL); U373 (IC_50_:7.2 μM); A549 (IC_50_:18 μM); OE21 (IC_50_:8.4 μM); SK-MEL-28 (IC_50_:21 μM); PC-3 (IC_50_: 8.1 μM); LoVo (IC_50_: 5.3 μM)	Smyrniotopoulos et al., 2008, 2010a
4*R*-Hydroxy-1-deoxy-bromotetrasphaerol	2010	Palaiokastritsa bay, Corfu Island, Greece	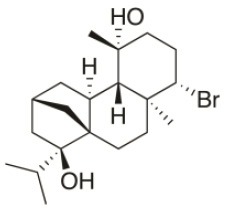	U373 (IC_50_:75 μM); A549 (IC_50_:63 μM); OE21 (IC_50_:64 μM); PC-3 (IC_50_: 43 μM); LoVo (IC_50_: 56 μM)	Smyrniotopoulos et al., 2010a,c
Coronone	2010	Palaiokastritsa bay, Corfu Island, Greece	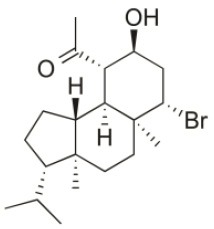	U373 (IC_50_:31 μM); A549 (IC_50_:42 μM); SK-MEL-28 (IC_50_:38 μM); OE21 (IC_50_:30 μM); PC-3 (IC_50_:30 μM); LoVo (IC_50_:28 μM)	Smyrniotopoulos et al., 2010a
Sphaerollane-I	2009	Palaiokastritsa bay, Corfu Island, Greece	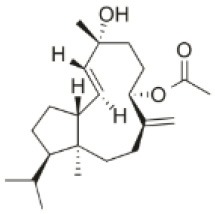	U373 (IC_50_:20 μM); A549 (IC_50_:44 μM); SK-MEL-28 (IC_50_:57 μM); OE21 (IC_50_:34 μM); PC-3 (IC_50_:34 μM); LoVo (IC_50_:23 μM)	Smyrniotopoulos et al., 2009, 2010a
Sphaerostanol	2010	Palaiokastritsa bay, Corfu Island, Greece	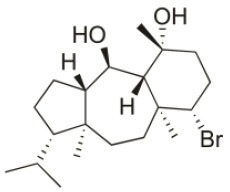	U373 (IC_50_:85 μM); A549 (IC_50_:97 μM); SK-MEL-28 (IC_50_:96 μM); OE21 (IC_50_:60 μM); PC-3 (IC_50_:74 μM); LoVo (IC_50_:64 μM)	Smyrniotopoulos et al., 2010a
10*R*-Hydroxy-bromocorodienol	2010	Palaiokastritsa bay, Corfu Island, Greece	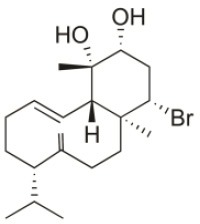	U373 (IC_50_:60 μM); A549 (IC_50_:64 μM); SK-MEL-28 (IC_50_:62 μM); OE21 (IC_50_:33 μM); PC-3 (IC_50_:48 μM); LoVo (IC_50_:24 μM)	Smyrniotopoulos et al., 2010a
Sphaerodactylomelol	2015	Berlenga Nature Reserve, Peniche, Portugal	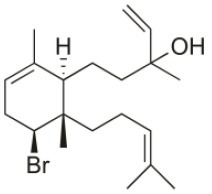	Inhibition of cell proliferation (IC_50_: 280 μM); Cytotoxicity (IC_50_: 720 μM) on HepG2 cells	Rodrigues et al., 2015
Spirosphaerol	2015	Liapades Bay, Corfu, Greece	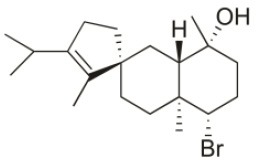	A549 (IC_50_:69 μM); Hs683 (IC_50_:56 μM); MCF-7 (IC_50_:67 μM); B16F10 (IC_50_:65 μM)	Smyrniotopoulos et al., 2015
Anthrasphaerol	2015	Liapades Bay, Corfu, Greece	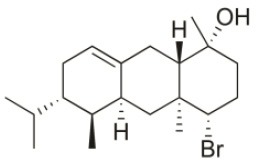	A549 (IC_50_:90 μM); Hs683 (IC_50_:93 μM); MCF-7 (IC_50_:85 μM); B16F10 (IC_50_:63 μM)	Smyrniotopoulos et al., 2015
Corfusphaeroxide	2015	Liapades Bay, Corfu, Greece	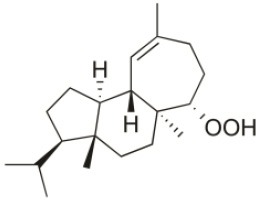	A549 (IC_50_:67 μM); Hs683 (IC_50_:63 μM); MCF-7 (IC_50_:60 μM); U373 (IC_50_:81 μM); SK-MEL-28 (IC_50_:75 μM); B16F10 (IC_50_:46 μM)	Smyrniotopoulos et al., 2015

* *Induce cytostatic activity on U373 cells inhibiting cell entrance into mitosis (3 μM). For the others compounds the possible intracellular signaling pathways were not characterized*.

## Conclusions and final remarks

Over the last several decades, marine organisms revealed to be an interesting source of both pre-existing and unrecognized compounds with the potential for providing sustainable economic and human benefits. Many of these compounds demonstrated great potential for therapeutic applications, exhibiting specific and potent activities against different diseases, including cancer. Their potential as a source of antitumor drugs has been proven by the current pipeline. Six of nine drugs from marine origin currently in the market are used in cancer treatment, and several compounds originated or derived from marine organisms are undergoing clinical trials with indications for oncologic therapeutics. However, the number of compounds in the market or in clinical trials is very low compared with the total number of isolated compounds with antitumor potential. This is mainly due to the different constraints between discovery and commercialization such as the discontinuation of “candidates” in clinical trials or preclinical tests due to difficulties on harvesting the organism and on the isolation and purification procedures, low yields, insufficient investment by pharmaceutical companies, environmental policies, high toxicity and low efficiency of the active compounds. Nevertheless, considering the approved marine drugs, it is also possible to observe that six of them were approved in the last 10 years. This fact is probably directly associated with the development of high-throughput screening technologies that allowed faster and more accurate results. Thus, it is expected that the number of antitumor drugs of marine origin will increase in the next few years.

Among the marine organisms, many crude extracts, enriched fractions and compounds obtained from algae displayed interesting antitumor potential along the years. These effects are mediated by compounds from different chemical classes including polysaccharides, terpenoids, phenolic compounds, glicoproteins, sterols, carotenoids, alkaloids, sulfolipids, chlorophylls, and fatty acids. Despite the high number of reports evidencing the cytotoxic and cytostatic properties of algae-derived compounds, few studies have characterized the intracellular signaling pathways underlying their effects. This view is clearly evident with *Sphaerococcus coronopifolius*, since some of its bioactive compounds exhibited interesting cytotoxic activities on different cell lines, but the intracellular signaling pathways involved in its activities have still not been deeply characterized. Thus, there is a need to design and perform studies to evaluate these type of molecules in more complex biological systems, including *in vivo* models. Another important tool that still remain unexplored is the use of 3D chemical structural modeling techniques to find new biochemical targets for the algae-derived compounds. Moreover, the idea of using algae-derived compounds as co-adjuvants in therapeutics should also be evaluated.

One of the major challenges in this area is the sustainable production of these compounds to “supply” sufficient quantity for preclinical, clinical and future commercialization, since the algae's slow growth and seasonality together with low extraction yields are significant limitations. In line with this requirement, total chemical synthesis can not only guarantee the sustainable and continuous production of the bioactive molecules but also improve or enhance their functional zones. Aquaculture can also be an interesting approach for the continuous supply of algae. However, more studies are needed to understand if algae continue to produce the desired compounds under artificial conditions.

The applications of new techniques such as nanotechnology to develop new nano-formulations can potentiate the development of anticancer drugs from algae origin leading to the suppression of limitations and improve the capabilities of some algae-derived compounds. However further technologies are needed to validate the improvement of their antitumor activities when applied in nanoformulations. According with this search this area was not yet explored. By other side, the creation/ establishment of interdisciplinary teams (including for example chemists, pharmaceuticals, biotechnologists and biologists) can contribute a faster bioscreening process, from the isolation and identification of algae-derived compounds to full validation of their antitumor capabilities. The studies gathered in the present review clearly demonstrate that algae have great potential as a source of antitumor compounds with the capacity to hamper different key pathway targets involved in cancer development including cell cycle regulation, apoptosis, angiogenesis, migration and invasion processes. Even though, to date algae have not been fully exploited, it is expected that in a few years, their translation to clinical use can be a reality.

## Author contributions

CA and RP conceived the research topic and the design the review. CA, JS, and SP did the bibliographic research. HG and MA critically contributed to discuss the cancer threat and current marine clinical pipeline. LB critically contributed to discuss the antitumor potential of marine algae-derived compounds. All authors performed the literature review and participated in the drafting and revision of the manuscript, thus making a direct and intellectual contribution to the work.

### Conflict of interest statement

The authors declare that the research was conducted in the absence of any commercial or financial relationships that could be construed as a potential conflict of interest.
